# Gene Expression Profiling in Limb-Girdle Muscular Dystrophy 2A

**DOI:** 10.1371/journal.pone.0003750

**Published:** 2008-11-18

**Authors:** Amets Sáenz, Margarita Azpitarte, Rubén Armañanzas, France Leturcq, Ainhoa Alzualde, Iñaki Inza, Federico García-Bragado, Gaspar De la Herran, Julián Corcuera, Ana Cabello, Carmen Navarro, Carolina De la Torre, Eduard Gallardo, Isabel Illa, Adolfo López de Munain

**Affiliations:** 1 Experimental Unit, Hospital Donostia, Donostia-San Sebastián, Basque Country, Spain; 2 Department of Computer Science and Artificial Intelligence, Computer Science Faculty, University of the Basque Country, San Sebastián, Basque Country, Spain; 3 Laboratoire de Biochimie et Génétique Moléculaire, Hôpital Cochin, Groupe Hospitalier Pitié-Salpêtrière, Paris, France; 4 Department of Pathology, Hospital Virgen del Camino, Pamplona, Spain; 5 Department of Orthopedic Surgery, Hospital Donostia, Donostia-San Sebastián, Basque Country, Spain; 6 Department of Pathology , Hospital 12 de Octubre, Madrid, Spain; 7 Department of Pathology , Hospital Meixoeiro, Vigo, Spain; 8 Laboratory of Experimental Neurology, Hospital de la Santa Creu i Sant Pau, Barcelona, Spain; 9 Department of Neurology, Hospital de la Santa Creu i Sant Pau, Barcelona, Spain; 10 Department of Neurology, Hospital Donostia, Donostia-San Sebastián, Basque Country, Spain; Hospital Vall d'Hebron, Spain

## Abstract

Limb-girdle muscular dystrophy type 2A (LGMD2A) is a recessive genetic disorder caused by mutations in calpain 3 (CAPN3). Calpain 3 plays different roles in muscular cells, but little is known about its functions or in vivo substrates. The aim of this study was to identify the genes showing an altered expression in LGMD2A patients and the possible pathways they are implicated in. Ten muscle samples from LGMD2A patients with in which molecular diagnosis was ascertained were investigated using array technology to analyze gene expression profiling as compared to ten normal muscle samples. Upregulated genes were mostly those related to extracellular matrix (different collagens), cell adhesion (fibronectin), muscle development (myosins and melusin) and signal transduction. It is therefore suggested that different proteins located or participating in the costameric region are implicated in processes regulated by calpain 3 during skeletal muscle development. Genes participating in the ubiquitin proteasome degradation pathway were found to be deregulated in LGMD2A patients, suggesting that regulation of this pathway may be under the control of calpain 3 activity. As frizzled-related protein (FRZB) is upregulated in LGMD2A muscle samples, it could be hypothesized that β-catenin regulation is also altered at the Wnt signaling pathway, leading to an incorrect myogenesis. Conversely, expression of most transcription factor genes was downregulated (MYC, FOS and EGR1). Finally, the upregulation of IL-32 and immunoglobulin genes may induce the eosinophil chemoattraction explaining the inflammatory findings observed in presymptomatic stages. The obtained results try to shed some light on identification of novel therapeutic targets for limb-girdle muscular dystrophies.

## Introduction

Limb-girdle muscular dystrophy type 2A (LGMD2A) is a recessive genetic disorder caused by mutations in calpain 3 (CAPN3), a muscle-specific, calcium-dependent cystein protease. Calpain 3 structure is similar to that of the ubiquitous calpains 1 and 2, but calpain 3 has specific regions (NS, IS1, and IS2) that confer it special characteristics such as autocatalytic and nuclear translocation capacity. Although calpain 3 was identified in 1989 [1], little is known about its function or its *in vivo* substrates. It has been reported to play different roles in the cell. Calpain 3 has a certain role in direct and indirect regulation of conventional calpains by proteolytic degradation of calpains and calpastatin respectively [2]. It may be involved in muscle contraction due to its link to titin and to its regulation by calcium [3]–[7].

Calpain 3 was shown to be in complex with dysferlin, suggesting a membrane homeostasis role of calpain 3 [8], and more recent studies demonstrated that AHNAK, a novel component of the dysferlin protein complex, serves as a direct substrate of calpain 3 in cell culture [9].

On the other hand, it has been confirmed that calpain 3 can cleave the C-terminal portion of FLNC *in vitro* and suggested that FLNC may be an *in vivo* substrate for calpain 3, functioning to regulate protein-protein interactions with sarcoglycans. Thus, calpain-mediated remodeling of cytoskeletal-membrane interactions, such as those occurring during myoblast fusion and muscle repair, may involve regulation of FLNC-sarcoglycan interactions [10].

Its presence in the nucleus has led to suggest that calpain 3 plays an important role in regulation of transcription factors indirectly controlling apoptotic processes [11], [12]. Recent studies reported that the antiapoptotic factor, cellular FLICE inhibitory protein (c-FLIP), is NF-κB dependent and is only expressed when CAPN3 is present [13]. However, other studies suggest that apoptosis may be secondary to muscle damage and inflammatory response [14].

Based on the observation of the C3 knockout (C3KO) mice, it has been suggested that calpain 3 is necessary for ubiquitination and acts upstream of the ubiquitination machinery [15].

Inflammatory cells have been detected in muscle tissue from patients with mutations in the CAPN3 gene in early stages [16] as happen in other distrophies. The role of inflammation in many dystrophies seems to be unexplained, and it has been related to the presence of signaling factors (cytokines) that withstand inflammatory mechanisms and regulatory phenomena [17]–[19].

In this study, the RNA expression profiling in muscle from biopsies of LGMD2A patients and control subjects were compared in order to determine the potential functions and the pathways in which calpain 3 is implicated.

## Materials and Methods

### Muscle samples and RNA processing

Muscle biopsies were taken from 10 LGMD2A patients (3 females and 7 males aged 13–48 years, mean age 29,5 years) and 10 controls (2 females and 8 males aged 22–84 years, mean age 50,2 years). Two out of the 10 LGMD2A patients showed an inflammatory pattern with eosinophilic infiltrates in their biopsies.

For diagnostic purposes deltoid, quadriceps, and biceps muscle specimens were collected using institutionally approved protocols and after obtaining informed consent (Table 1). Muscle tissues were snap frozen and stored at −80°C. Most of the 7 symptomatic cases showed similar necrosis and regenerating phenomena (data not available in one case).

**Table 1:** Distribution of muscle biopsies taken from 10 LGMD2A patients and 10 control subjects.

Biopsy Number	Status	Gender	Muscle	Age	Myopathological data	Gardner-Medwin-Walton Scale	CAPN3 mutations	
							Mutation 1	Mutation 2
EXP-01	LGMD2A	M	Quadriceps	34	Mild myopathic changes (fiber size alteration, centrally located nuclei and splitting) No necrosis, no regeneration, no lobulated fibers.	2	p.(Gly222Arg)	p.(Arg748Gln)
EXP-02	LGMD2A	F	Deltoid	33	Necrosis and regenerating phenomena	3	c.946-1G>A	p.(Gln660Arg)
EXP-03	LGMD2A	M	Quadriceps	37	Necrosis, regenerating phenomena and fibrosis	Unknown	p.(Met248Arg)	p.(Arg769Gln)
EXP-04	LGMD2A	M	Quadriceps	44	Necrosis and regenerating phenomena	Unknown	p.(Gln300X)	p.(Gln660Arg)
EXP-05	LGMD2A	M	Deltoid	13	Inflammatory reaction around necrotic and non necrotic fibers. Inflammation collects at endomysial site sometimes with perivascular infiltrate without destruction of walls of arterioles and venules. Numerous eosinophilic leucocytes are present.	Asymptomatic	p.(Arg788SerfsX14)	p.(Arg788SerfsX14)
EXP-09	LGMD2A	F	Biceps braquialis	14	Myositis with local infiltration of eosinophils. Patchy, focal inflammatory cell infiltrate with minor changes in the architecture of fibers without changes in the distribution of the fiber type.	Asymptomatic	p.(Arg490Trp)	p.(Gly691TrpfsX7)
EXP-35	LGMD2A	M	Deltoid	48	Necrosis, fibrosis, lobulated fibres	7	p.(Gln142X)	p.(Gln142X)
EXP-36	LGMD2A	M	Quadriceps	26	Necrosis and regenerating phenomena	2	p.(Lys254Glu)	c.1910delC
EXP-40	LGMD2A	M	Quadriceps	29	Mild myopathic changes (centrally located nuclei and fibrosis)	7	p.(Ala160Gly)	c.1029+3A>G
EXP-41	LGMD2A	F	Deltoid	17	No data available	2	c.2185-12_2194del	p.(Arg788SerfsX14)
EXP-25	Control	F	Deltoid	57				
EXP-27	Control	M	Quadriceps	50				
EXP-28	Control	M	Quadriceps	22				
EXP -29	Control	F	Quadriceps	73				
EXP -30	Control	M	Quadriceps	84				
EXP-31	Control	M	Quadriceps	46				
EXP-32	Control	M	Quadriceps	48				
EXP-33	Control	M	Deltoid	51				
EXP-38	Control	M	Quadriceps	31				
EXP-39	Control	M	Quadriceps	41				

The quality of all RNAs obtained from muscle biopsies (RNAPlus, QBiogene) was verified using spectrophotometry and the Bioanalyzer system (Agilent). All of them showed acceptable quality and integrity (RIN above 7) to be eligible for the experiment.

All RNAs were reverse-transcribed, and biotinylated cRNA probes were generated by *in vitro* transcription (Ambion, CA, USA). Fragmented cRNA of each sample was hybridized individually to human HG-U133A (22.283 probe sets) and HG-U133B (22.645 probe sets) GeneChips (Affymetrix, Santa Clara, California) in order to analyze the expression of 44.928 probes, comprising more than 33.000 genes.

### Data analysis

In-depth quality controls were performed to analyze the validity of the hybridization processes in accordance with four criteria. First, the correct presence of the signal corresponding to the spike control BioB. Second, the expression ratio between the 3′ and 5′ ends of the housekeeping GAPDH should not exceed a value of three. Third, the full percentage of presences detected by the Affymetrix Detection algorithm for each array must be in the range 40–60. And finally, the percentage of outlier probe sets detected within each microarray should be less than 5%. All hybridized arrays on the study met all four quality criteria, demonstrating the reliability of data generated.

The hybridized arrays were scanned, and raw data were extracted using the Microarray Analysis Suite 5.0 (MAS5; Affymetrix). The raw data were normalized using RMA (Robust Multichip Average) expression summary in Bioconductor [20]. RMA consists of three steps: a background adjustment, quantile normalization, and finally summarization [21]–[23].

The sensitivity of microarray-generated data to noise from experimental variables is well documented [24]. For the analysis, the average values of each tested group (patients and controls) were used in order to obtain the most homogeneous results, trying to avoid variability between individual cases due to different characteristics (genetic background, sex, age, muscles, mutations, etc.). Two statistical methods were applied in order to distinguish significant and substantial differential expression from noise and variation due to either genetic heterogeneity or experimental procedures.

First, in order to identify significantly different genes between LGMD2A patients and normal controls, a geometric fold-change analysis was used [24], [25]. The threshold was set at a two-fold change value. Using the criterion of fold-change implies that larger fold changes are most likely to be real and no hypothesis is assumed. Principal component analysis (PCA) was performed after array normalization. PCA is a technique that summarizes a large set of variables in a smaller set that retains the essential variance of the original data set [26]. PCA derives an equivalent, uncorrelated set of new variables from the original set of correlated variables according to their contribution to a ranked set of principal components [27].

Second, Class Comparison Difference Analyses were performed using BRB-ArrayTools developed by Dr. Richard Simon and BRB-ArrayTools Development Team. In order to identify probe sets with significant intensity differences between disease classes, a two-sample univariate *t*-test was applied to the unaffected control data set vs. the LGMD2A data set. The use of p-values implies hypothesis testing. It is assumed in the null hypothesis that there is no fold change and then evidence was looked for to reject it using a type-1 error. The threshold was set at p 0.001.

To minimize false positives, only the probe sets commonly yielded by both methodologies were included into the final list of genes differentially expressed in LGMD2A.

Moreover, as an additional supporting process, two machine learning feature selection techniques were run. Symmetrical uncertainty ranking [28] was first applied as an univariate criterion to measure the worth of each probe set alone: this computes the mutual information with respect to the class phenotype and compensates for the bias of the information gain. Correlation-based Feature Subset (CFS) selection [29], a multivariate feature selection that evaluates the merit of a probe set subset by measuring the individual predictive power of each probe set along with the redundancy within that subset, was then used. CFS outputs a subset of features instead of individual relevances.

The same procedure was used to compare samples from patients who were asymptomatic but had eosinophilic infiltrates in their muscle biopsies (2 cases) and samples from healthy controls (10 cases).

Microarray data have been submitted to the GEO (Gene Expression Omnibus) public database (accession GSE11681).

### Quantitative Real-Time PCR

To investigate the validity of array data, expression levels of the differentially expressed genes were measured using the TaqMan quantitative RT-PCR assay. Relative expression levels initially determined with the cDNA microarrays were correlated to the expression levels assessed using quantitative RT-PCR for each patient sample.

Whereas microarrays identify target genes of interest among thousands of genes, truly quantitative information relies on quantitative RT-PCR. Some of the significantly regulated changes found on the microarray could be replicated by quantitative RT-PCR. Quantitative RT-PCR was performed using the 7900 HT Fast Real-Time PCR System (Applied Biosystems). Because of the limiting RNA amount isolated from muscle biopsies used for microarray analysis, only a few samples (6 cases) were used for confirmation with quantitative RT-PCR experiments.

The TaqMan Low Density Arrays (TLDA) were purchased from Applied Biosystems, and the protocol recommended by the manufacturer was used. Customer-designed TLDAs were used in order to test a series of 63 genes. In order to select these genes, genes with unknown function, hypothetical proteins, and open reading frame regions were excluded. Gene families were represented including only some of the members, such as collagens, etc. Moreover, genes showing differential expression profiling in the comparison between patients with eosinophilic infiltrates and healthy controls were included in the TLDA, as well as genes with expression variation in other studies.

Expression levels for all transcripts were determined relative to the internal housekeeping control gene GAPDH in the TLDAs which, as expected, did not demonstrate altered expression according to microarray analysis.

In order to identify probe sets with significant intensity differences, the method applied to the unaffected control data set vs. the LGMD2A data set was Benjamini-Hochberg method using Stat Miner program (Integromics).

## Results

After having adjusted the background, normalized and summarized the data, the fold change obtained generates a list by magnitude of response. As a result of this method, the fold change analysis identified 156 differentially expressed probe sets in LGMD2A skeletal muscle compared to control skeletal muscle. Of these, 92 were significantly overexpressed and 64 showed a reduced expression in LGMD2A patients compared to the unaffected controls.

**Figure 1:**
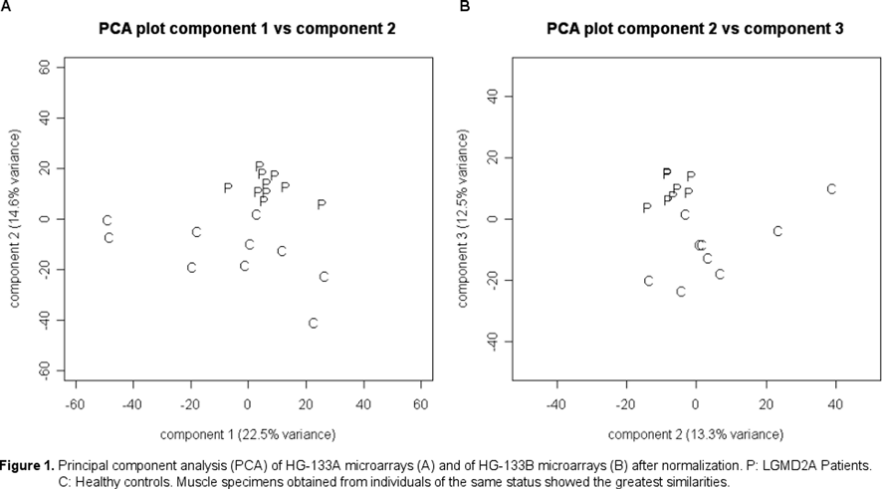
Principal component analysis (PCA) of HG-U133A microarrays (A) and of HG-U133B microarrays (B) after normalization. P: LGMD2A Patients. C: Healthy controls. Muscle specimens obtained from individuals of the same status showed the greatest similarities.

PCA grouped together on the one hand patient samples and on the other hand control samples and a greater variability was seen among controls due to the heterogeneity of this group (Figure 1).

On the other hand, the additional statistical method used to analyze the data, the Class Comparison Differences method, applied a two-sample univariate *t*-test to the unaffected control data set vs. the LGMD2A data set. This method identified 627 probe sets with a p value higher than 0.001.

However, the final list of genes comprised 86 probe sets (74 genes) commonly yielded by the two methodologies which were differentially expressed in LGMD2A compared to unaffected muscle biopsies. Of these 74 genes, 53 were overexpressed and 21 had a reduced expression in the LGMD2A patients and all the genes were clustered into functional groups (Table 2). Transcripts were classified according to different biological processes, as obtained from LocusLink (www.ncbi.nlm.nih.gov/LocusLink/): extracellular matrix proteins/phosphate transport, cell adhesion, muscle development, transcription factors, signaling pathways, metabolic process, transport, ubiquitin cycle, and other functions.

**Table 2:** Significantly differentially regulated transcripts, NV: Not validated by TLDAs.

Affymetrix ID	Biological process/Gene title	Gene symbol	Fold change	Parametric p value Class Comparison	Fold change Validated by RT-PCR	Significant p value Stat Miner
Extracellular matrix proteins						
202310_s_at	collagen, type I, alpha 1	COL1A1*	4.71	0.0003482	5.86	0.012178774
202404_s_at	collagen, type I, alpha 2	COL1A2*	4.72	6.61e-05	NV	–
211161_s_at	collagen, type III, alpha 1	COL3A1*	7.72	1.1e-06	13.71	–
201852_x_at			5.12	7.4e-06		
215076_s_at			4.79	3.48e-05		
212488_at	collagen, type V, alpha 1	COL5A1	2.07	8.18e-05	9.24	1.13E-04
221729_at	collagen, type V, alpha 2	COL5A2	3.12	<1e-07	NV	–
221730_at			2.12	3e-07		
203477_at	collagen, type XV, alpha 1	COL15A1*	2.80	2.36e-05	NV	–
225681_at	collagen triple helix repeat containing 1	CTHRC1	2.96	3.74e-05	NV	–
Cell adhesion						
201005_at	CD9 antigen (p24)	CD9 *	2.38	6.36e-05	2.16	5.71E-02
212063_at	CD44 antigen (homing function and Indian blood group system)	CD44 *	2.35	0.0002703	2.23	0.3071496
211719_x_at	fibronectin 1	FN1*	2.33	0.0006326	3.30	1.16E-02
210495_x_at			2.30	0.0001008		
216442_x_at			2.21	0.0001307		
Muscle development						
205940_at	myosin, heavy polypeptide 3, skeletal muscle, embryonic	MYH3*	11.78	1.4e-06	40.62	2.15E-04
205145_s_at	myosin, light polypeptide 5, regulatory	MYL5	4.28	3.49e-05	7.21	1.83E-02
204173_at	myosin light chain 1 slow a	MLC1SA ( = MYL6B)	2.41	0.0003024	3.28	1.77E-02
219829_at	integrin beta 1 binding protein (melusin) 2	ITGB1BP2	2.23	1.3e-06	2.50	0.013391304
Transcription factors						
202431_s_at	v-myc myelocytomatosis viral oncogene homolog (avian)	MYC*	0.48	0.0006587	0.35	–
201466_s_at	v-jun sarcoma virus 17 oncogene homolog (avian)	JUN*	0.47	4.98e-05	0.69	0.443749559
219990_at	E2F transcription factor 8	E2F8	3.22	0.0001105	4.52	4.80E-03
201473_at	jun B proto-oncogene	JUNB*	0.41	0.0002329	NV	–
209189_at	v-fos FBJ murine osteosarcoma viral oncogene homolog	FOS*	0.12	7.52e-05	0.10	–
203973_s_at	CCAAT/enhancer binding protein (C/EBP), delta	CEBPD*	0.39	0.0009174	1.61	0.266597408
201694_s_at	early growth response 1	EGR1*	0.31	5.16e-05	0.13	1.74E-03
227404_s_at			0.12	8.11e-05		
209357_at	Cbp/p300-interacting transactivator, with Glu/Asp-rich carboxy-terminal domain, 2	CITED2	0.39	9e-07	0.57	0.255171653
210479_s_at	RAR-related orphan receptor A	RORA	0.46	0.0003675	NV	–
202393_s_at	Kruppel-like factor 10	KLF10	0.45	0.0000651	0.49	0.130862236
221778_at	KIAA1718 protein	KIAA1718 (JHDM1D)	0.48	0.0009927	NV	–
Signal transduction						
209541_at	insulin-like growth factor 1 (somatomedin C)	IGF1*	2.81	1e-07	1.96	7.01E-02
209822_s_at	very low density lipoprotein receptor	VLDLR*	2.63	3e-07	NV	–
213880_at	leucine-rich repeat-containing G protein-coupled receptor 5	LGR5	0.30	0.0004577	0.23	6.35E-02
219654_at	protein tyrosine phosphatase-like (proline instead of catalytic arginine), member a	PTPLA	2.42	4.83e-05	NV	–
222918_at	RAB9B, member RAS oncogene family	RAB9B	2.04	1.09e-05	NV	–
212099_at	ras homolog gene family, member B	RHOB	0.41	0.0000594	NV	–
217728_at	S100 calcium binding protein A6 (calcyclin)	S100A6*	2.26	0.0008602	2.43	5.00E-02
Signal pathways						
200665_s_at	secreted protein, acidic, cysteine-rich (osteonectin)	SPARC*	2.02	0.000903	2.55	5.82E-02
218087_s_a	sorbin and SH3 domain containing 1	SORBS1	2.05	0.000123	NV	–
214844_s_at	docking protein 5	DOK5	2.24	3.16e-05	3.16	6.09E-03
203697_at	frizzled-related protein	FRZB	5.42	0.000466	13.16	2.41E-04
203698_s_at			2.99	0.000466		
203789_s_at	sema domain, immunoglobulin domain (Ig), short basic domain, secreted, (semaphorin) 3C	SEMA3C	2.86	0.0001054	NV	–
201309_x_at	chromosome 5 open reading frame 13	C5orf13	2.09	0.0005666	NV	–
236860_at	Neuropeptide Y receptor Y6 (pseudogene)	NPY6R	4.09	0.0007927	NV	–
Metabolic process						
201425_at	aldehyde dehydrogenase 2 family (mitochondrial)	ALDH2	0.43	8.62e-05	0.67	0.459523739
209301_at	carbonic anhydrase II	CA2*	0.48	0.0007153	0.79	0.522535838
202464_s_at	6-phosphofructo-2-kinase/fructose-2,6-biphosphatase 3	PFKFB3	0.15	1.5e-06	0.45	0.141846652
Transport						
208691_at	transferrin receptor (p90, CD71)	TFRC*	8.11	6.47e-05	4.52	4.80E-03
207332_s_at			5.99	6.28e-05		
204430_s_at	solute carrier family 2 (facilitated glucose/fructose transporter), member 5	SLC2A5	2.17	0.0003736	3.74	2.69E-03
201560_at	chloride intracellular channel 4	CLIC4	2.20	0.0001832	NV	–
202236_s_at	solute carrier family 16 (monocarboxylic acid transporters), member 1	SLC16A1	2.55	<1e-07	NV	–
205073_at	Cytochrome P450, family 2, subfamily J, polypeptide 2	CYP2J2	2.04	3.4e-05	NV	–
224579_at	Solute carrier family 38, member 1	SLC38A1	2.58	0.0007761	NV	–
219525_at	hypothetical protein FLJ10847	FLJ10847 (SLC47A1)	0.47	6.42e-05	NV	–
217966_s_at	chromosome 1 open reading frame 24	C1orf24 (FAM129A)	0.47	0.00045	0.78	0.55888133
Ubiquitin cycle						
218306_s_at	hect (homologous to the E6-AP (UBE3A) carboxyl terminus) domain and RCC1 (CHC1)-like domain (RLD) 1	HERC1	2.22	0.0000045	2.45	2.29E-02
218575_at	Anaphase promoting complex subunit 1	ANAPC1	2.02	9e-07	1.31	0.447268323
229267_at			2.07	0.0001629		
Other functions						
218273_s_at	protein phosphatase 2C, magnesium-dependent, catalytic subunit (mitochondrial)	PPM2C	2.49	1.55e-05	3.08	2.97E-02
201609_x_at	isoprenylcysteine carboxyl methyltransferase	ICMT*	2.25	7.2e-06	NV	–
201611_s_at			2.00	1.88e-05		
202965_s_at	calpain 6	CAPN6	2.05	1.71e-05	5.31	9.12E-04
212848_s_at	chromosome 9 open reading frame 3	C9orf3	2.01	0.0003013	NV	–
201010_s_at	Thioredoxin interacting protein	TXNIP*	0.43	1e-05	0.49	0.133790906
201009_s_at			0.42	0.0009904		
202917_s_at	S100 calcium binding protein A8 (calgranulin A)	S100A8	0.34	0.0002179	0.27	1.51E-02
209398_at	histone 1, H1c	HIST1H1C	0.45	0.0003089	0.76	0.491015255
225061_at	DnaJ (Hsp40) homolog, subfamily A, member 4	DNAJA4*	2.53	0.0000529	3.65	8.54E-03
209596_at	matrix-remodelling associated 5	MXRA5	2.90	5e-07	NV	–
219087_at	asporin (LRR class 1)	ASPN	4.58	4e-07	NV	–
235022_at	chromosome 18 open reading frame 19	C18orf19	2.34	1e-07	NV	–
218820_at	chromosome 14 open reading frame 132	C14orf132	2.14	2.87e-05	NV	–
202016_at	mesoderm specific transcript homolog (mouse)	MEST	2.11	0.000709	NV	–
218999_at	hypothetical protein FLJ11000	FLJ11000 (TMEM140)	0.49	218999_at	NV	–
224836_at	tumor protein p53 inducible nuclear protein 2	TP53INP2*	2.46	0.0003853	NV	–
Unknown function						
238124_at	Myomesin family, member 3	MYOM3	2.30	0.0000126	6.74	1.86E-04
230284_at			2.01	0.0000003		
202759_s_at	PALM2-AKAP2 protein	PALM2-AKAP2	2.16	0.0000007	NV	–
229778_at	Hypothetical protein MGC10946	MGC10946 (C12orf39)	2.75	0.0000022	NV	–
211071_s_at	myeloid/lymphoid or mixed-lineage leukemia (trithorax homolog, Drosophila); translocated to, 11	MLLT11	3.92	0.0002385	NV	–
218876_at	brain specific protein	CGI-38 (TPPP3)	2.86	0.0000867	NV	–
221104_s_at	nipsnap homolog 3B (C, elegans)	NIPSNAP3B	2.68	0.0001963	NV	–
225242_s_at	steroid sensitive gene 1	URB (CCDC80)	3.22	0.0004132	NV	–

* Dysregulated genes in FSHD, DMD, α-sarcoglycan, and congenital myopathies (Campanaro et al 2002, Winokur et al 2003, Haslett et al 2003, Taniguchi et al 2006, Osborne et al 2007).

The additional supporting process, the Correlation-based Feature Subset selection (CFS) [29] highlighted a set of 21 genes. Of these 21 genes, 7 corresponded to the previously determined group of 74 genes. In turn, Symmetrical Uncertainty Ranking returned correlation coefficients higher than 0.5 for 24 genes within the list. Note that the highest correlation was 0.816 when the coefficient was constrained between 1 (maximum) and 0 (minimum). The average coefficient for the whole gene list was 0.36, with a standard deviation of 0.177.

### Overview of expression profiling in LGMD2A muscles

Some transcript classes were of particular interest in this analysis (Table 2). Most genes found to be dysregulated in LGMD2A were genes grouped in the transcription factor category, and some of them showed the lowest fold-change values obtained in the study (FOS, EGR1). By contrast, genes showing the highest fold-change values included extracellular matrix proteins, genes involved in muscle development, and three additional genes with different functions (FRZB, TFRC, and CAPN6).

As a whole, in most of the biologically classified processes, the same trend to up- or downregulation was seen for all genes involved in the same process. Genes associated with extracellular matrix (collagen types I, III, V, and XV, and SPARC), cell adhesion, muscle development (MYH3, MYL5 and ITGB1BP2), signaling pathways, and ubiquitin cycle predominated among upregulated genes. However, all genes involved in metabolic processes and transcription factors (except for the E2F8 gene) were downregulated (Table 2).

On the other hand, upregulation of IGF1, which is a regulator of somatic growth and cell proliferation, was seen in this study. IGFa is an inducer of different pathways such as the phosphatidylinositol 3-kinase survival (through activation of AKT1, AKT2), the calcineurin.mediated signaling pathways, and of GATA2 activation.

HERC1 and ANAPC1 are genes implicated in the ubiquitin cycle and showed upregulation in LGMD2A muscle samples. HERC1, ubiquitously expressed, is located in the cytosol in the Golgi apparatus, stimulating guanine nucleotide, forming a cytosolic ternary complex with clathrin and Hsp70, and is involved in protein trafficking. ANAPC1 is a component of the anaphase promoting complex/cyclosome (APC/C), a cell cycle-regulated E3 ubiquitin ligase that controls progression through mitosis and the G1 phase of the cell cycle.

There are two deregulated genes according to our results whose cell location is the mitochondrion matrix, one of which is involved in the metabolic process, ALDH2 (aldehyde dehydrogenase 2 family) (downregulated), while the other, the PPM2C gene (protein phosphatase 2C, magnesium-dependent, catalytic subunit) (upregulated) is implicated in protein amino acid dephosphorylation.

### Expression changes in common with other muscular dystrophies

Twenty four out of the 74 deregulated genes with altered expression in LGMD2A were also deregulated in other muscular dystrophies (DMD, α-SGD, FSHD, dysferlinopathies, Fukuyama-type congenital muscular dystrophy, and laminin-α2 deficient congenital muscular dystrophy) [24], [30]–[35] (Table 2).

### LGMD2A and eosinophil infiltration

A comparison was made between biopsies of control muscles (10 cases) and biopsies from two cases showing eosinophil infiltrates. Results of this comparison are summarized in Table 3.

**Table 3:** Significantly differentially regulated transcripts comparing patients with eosinophilic infiltrates to healthy controls.

Probe set	Biological process/Gene title	Gene symbol	Fold change	Parametric p-value
Phosphate transport				
212489_at	collagen, type V, alpha 1	COL5A1	2.39	0.0000009
221729_at	collagen, type V, alpha 2	COL5A2	3.20	0.0001189
225681_at	collagen triple helix repeat containing 1	CTHRC1	3.25	0.0002197
230867_at	hypothetical protein LOC131873	LOC131873	2.28	0.0000307
Signaling pathway				
203697_at	frizzled-related protein	FRZB	7.67	0.0004106
203698_s_at			3.02	0.0004541
Signal transduction				
209541_at	insulin-like growth factor 1 (somatomedin C)	IGF1	3.06	0.0000059
209542_x_at			2.51	0.000116
211577_s_at			2.14	0.0001273
209822_s_at	very low density lipoprotein receptor	VLDLR	3.35	0.0002441
Immune response				
203828_s_at	interleukin 32///interleukin 32	IL32	5.45	0.0005892
211430_s_at	immunoglobulin heavy locus///immunoglobulin heavy constant gamma 1 (G1m marker)///immunoglobulin heavy constant gamma 2 (G2m marker)///immunoglobulin heavy constant gamma 3 (G3m marker)///immunoglobulin heavy constant mu	IGHIGHG1IGHG2IGHG3IGHM	4.37	0.0001606
221651_x_at	immunoglobulin kappa constant///immunoglobulin kappa variable 1-5	IGKC///IGKV1-5	2.21	0.0001881
Transcription				
214608_s_at	eyes absent homolog 1 (Drosophila)	EYA1	2.30	0.0001005
209357_at	Cbp/p300-interacting transactivator, with Glu/Asp-rich carboxy-terminal domain, 2	CITED2	0.34	0.0009738
227705_at	transcription elongation factor A (SII)-like 7	TCEAL7	2.79	0.000246
Metabolic process				
43427_at	acetyl-Coenzyme A carboxylase beta	ACACB	0.44	0.0002438
49452_at			0.39	0.0001128
224918_x_at	microsomal glutathione S-transferase 1	MGST1	0.27	0.0003928
Transport				
202236_s_at	solute carrier family 16 (monocarboxylic acid transporters), member 1	SLC16A1	2.74	0.0000816
239984_at	sodium channel, voltage-gated, type VII, alpha	SCN7A	2.64	0.0005832
Other functions				
205206_at	Kallmann syndrome 1 sequence	KAL1	0.25	0.0004374
226312_at	TORC2-specific protein AVO3	AVO3	0.47	0.0003421
227013_at	LATS, large tumor suppressor, homolog 2 (Drosophila)	LATS2	0.41	0.0005208
224842_at	PI-3-kinase-related kinase SMG-1	SMG1	0.39	0.0002757
202965_s_at	calpain 6	CAPN6	4.00	<1e-07
201609_x_at	isoprenylcysteine carboxyl methyltransferase	ICMT	2.35	0.0007576
202998_s_at	lysyl oxidase-like 2	LOXL2	2.03	0.0004611
209596_at	matrix-remodelling associated 5	MXRA5	4.66	0.0001074
209398_at	histone 1, H1c	HIST1H1C	0.26	0.0007007
226322_at	ARG99 protein ( Gene name: TMTC1)	ARG99	0.45	0.0002993
205381_at	leucine rich repeat containing 17	LRRC17	2.60	0.0001683
219087_at	asporin (LRR class 1)	ASPN	5.76	0.000238
Unknown				
202760_s_at	A kinase (PRKA) anchor protein 2///PALM2-AKAP2 protein	AKAP2///PALM2-AKAP2	2.71	0.0000528
202759_s_at	PALM2-AKAP2 protein	PALM2-AKAP2	3.29	0.0000042
226155_at	KIAA1600	KIAA1600	0.46	0.0004366
235022_at	chromosome 18 open reading frame 19	C18orf19	2.46	0.0002359
229778_at	hypothetical protein MGC10946	MGC10946	2.92	0.0007268
219525_at	hypothetical protein FLJ10847	FLJ10847	0.42	0.0004347
211724_x_at	hypothetical protein FLJ20323	FLJ20323	0.45	0.0003093
225893_at	clone TESTIS-724 mRNA sequence		0.41	0.0001744

Genes involved in immune response such as IL-32, IGHG1, and IGKC were upregulated, as well as CAPN6, while those involved in chemotaxis or regulation of the protein kinase B signaling cascade were downregulated in asymptomatic cases comparing with controls.

### Validation of microarray data by quantitative RT-PCR

**Figure 2:**
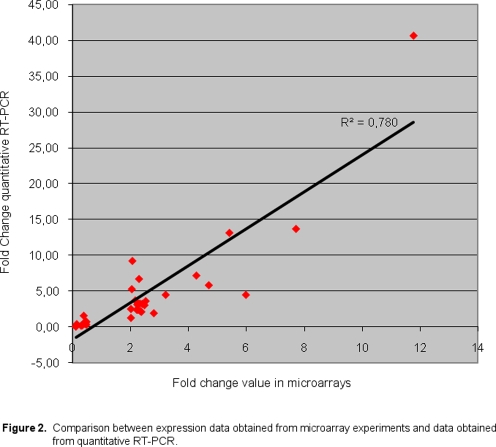
Comparison between expression data obtained from microarray experiments and data obtained from quantitative RT-PCR.

The main correlation between the two assays is showed in Figure 2. Real-time PCR was not only used to confirm the abnormal gene expression profiles detected by microarray analysis, but also to better define fold-change variations using a more sensitive approach. This approach showed mean fold changes in expression levels directionally similar to those determined by microarray analysis. Overall, fold change was lower when the microarray approach was used compared to real-time PCR (Table 2).

## Discussion

The main correlation between the two assays, microarrays and quantitative RT-PCR, was high for all genes, indicating a good agreement between both assays for identification of deregulated genes.

On the other hand, by means of the additional supporting process, a high correlation degree among results was shown and it provided more reliability to the final list of genes. Therefore, results of the two machine learning approaches support the degree of relevance of the 74 genes identified.

Calpain 3 was not abnormally regulated in the microarray study (data not shown). While protein analysis usually shows an absence of protein in patients, the microarray data did not reveal a reduction of calpain 3 mRNA indicating that this primary genetic defect cannot be identified by expression profiling. It is worthwhile mentioning that the presence of different missense mutations in most of the patients may explain this observation as well as the variability found in the Western Blot including normal patterns [36].

In LGMD2A muscles, genes associated with ECM/membrane-related, cell adhesion genes, muscle development genes, signaling pathway genes, and ubiquitin cycle genes were upregulated (Table 2).

### Extracellular matrix

The general trend for structural genes to be expressed at higher levels in patients could reflect a general upregulation of structural genes in the mutant muscle, as previously reported for other types of muscular dystrophies [37].

It is interesting to note that a large proportion of genes associated to the extracellular matrix were probably upregulated as a result of fibrotic infiltration. These genes include extracellular matrix proteins such as collagen types I and III (the two major collagens in the ECM), cell adhesion proteins such as CD9, CD44, and fibronectin.

SPARC, overexpressed in the fibroblasts of skin biopsy specimens obtained from patients with systemic sclerosis [38], could be the factor involved in the interstitial fibrosis seen in muscles of LGMD2A patients. It is a matricellular glycoprotein that may modulate cell interaction within the ECM by binding to both ECM structural components and growth factors.

### Muscle development

Our results showed that MYH3 (myosin, heavy chain 3, skeletal muscle, embryonic) is highly upregulated in samples from LGMD2A patients. Expression of embryonic myosin heavy chain is a hallmark of muscle regeneration after birth and a characteristic marker of human muscular dystrophies. During normal human development, expression is restricted to the embryonic period of development [39]. This could indicate a failed muscular regeneration attempt to compensate a downstream injury.

Another upregulated gene is myosin light chain 1 slow A (MLC1SA), a transcriptional regulator promoting muscle cell proliferation expressed in both slow-twitch skeletal muscle and non-muscle tissue. This gene showed a high individual correlation with class phenotype (0.442), and was one of the seven genes included in the CFS output. This fact flawlessly demonstrates its importance not only from an individual point of view, but also because of its potential interactions. MLC1SA is one of the two phosphorylable regulatory light chains forming the myosin complex. Cohen et al [40] found that MLC1SB (Accession N° P09542 in mice) was a substrate for calpain 3. To date, no contractile proteins have been identified as *in vivo* substrates for CAPN3. Identification of MLC1 as a potential substrate for CAPN3 was of interest because, in a previous study, Kramerova et al [15] demonstrated that CAPN3 regulates sarcomere remodeling by acting upstream of the ubiquitin–proteasome system.

LGMD2A patients showed upregulation of the IGF-1 gene as previously observed in other muscular dystrophies. Normal skeletal muscle is able to efficiently repair itself in response to injury. IGF-1 has been implicated as a factor that may affect many steps in gene expression control, including cell proliferation, differentiation, and degradation processes. IGF-1 is a peptide that has been shown to have anabolic effects on muscle cells. This action can be explained based on the molecular signaling events initiated by its receptor, a tyrosine kinase activated on IGF-1 binding, and transmitted through a cascade of intracellular events, leading to a general increase in protein synthesis [41], [42].

Integrin β1 binding protein (ITGB1BP2 = melusin), also upregulated in LGMD2A muscles, is present in a costamere-like pattern consisting of two rows flanking a-actinin at Z line. Melusin expression is upregulated during *in vitro* differentiation of the C2C12 murine myogenic cell line, and is regulated during *in vivo* skeletal muscle development [43]. Upregulation of the melusin gene may alter a process that is tightly controlled in muscle development, leading to inadequate muscle differentiation and maturation. The generalized inhibition of terminal stages of myogenic differentiation in C3KO myotubes affects at least two events: sarcomere formation and integrin isoform replacement [44]. During myogenesis, two isoforms of β1 integrin are expressed: β1A is expressed in myoblasts and is downregulated during myogenesis, while β1D appears after fusion and eventually displaces β1A in mature myotubes [45]. Neither β1A nor β1D were cleaved by CAPN3, suggesting that changes in the level of integrin isoforms are not a direct result of calpain 3 absence [44].

### Ubiquitin cycle and protein degradation

It is still unclear whether CAPN3 directly cleaves proteins to make them available for ubiquitination or whether the effect of CAPN3 is indirect (i.e. through regulation of other proteins involved in ubiquitination) [15]. In LGMD2A muscle samples, the HERC1 and ANAPC1 genes involved in the ubiquitin cycle are upregulated, suggesting that their regulation may be under the control of calpain 3.

Moreover, Ono et al [46] found proteolysis of proteasome regulatory subunit RPS6A by calpain 3, which may indicate that the ubiquitin-proteasome system is subject to regulation by calpain.

As ubiquitination tags proteins for degradation, decreased ubiquitination may lead to excessive accumulation of the proteins that should otherwise be degraded. This in turn could trigger a cell stress response, one manifestation of which is upregulation of heat shock proteins [15]. According to the reported data, the DnaJ (Hsp40) homolog, subfamily A member 4 (DNAJA4), that showed upregulation, may regulate the chaperone function of Hsp70 proteins [47].

### Signaling pathways

The protein coded by HERC1, upregulated in LGMD2A patients, has a C-terminal HECT (homologous to E6-AP C-terminus) domain, which suggests that it has an ubiquitin ligase activity.

β-catenin plays a critical role in many cellular and morphogenic processes by performing two distinct functions: in the nucleus, it acts as a mandatory coactivator of TCF/LEF transcription factors in response to Wnt signaling during both embryonic development and adult muscle regeneration, while at the cell membrane, β-catenin associates with the cadherin complex that links adhesion molecules to the cytoskeleton. In both cases, the concentration of β-catenin has been shown to be tightly regulated through ubiquitin-mediated degradation [44].

Two distinct ubiquitin ligase complexes control β-catenin levels in cytoplasm and at the membrane [48]. Ubiquitination and degradation of the cytosolic pool of β-catenin are under the control of Wnt signaling. Degradation of the membrane pool of β-catenin in skeletal muscle is mediated by the Ozz-E3 ubiquitin ligase complex [49]. Thus, it may be suggested that membrane β-catenin is indirectly regulated by CAPN3. It should also be noted that Trim32, found mutated in limb-girdle muscular dystrophy type 2H, is another putative E3-ubiquitin-ligase [50].

On the other hand, frizzled-related protein (FRZB) is upregulated in LGMD2A muscle samples. It could therefore be hypothesized that β-catenin regulation is also altered at the Wnt signaling pathway, leading to an abnormal myotube fusion or incorrect myogenesis.

### Deregulation of mitochondrial genes

In our results, the mitochondrial genes found to be deregulated were ALDH2 and PPM2C. ALDH2 was downregulated in patient samples and is implicated in the glycolysis/gluconeogenesis pathway. On the contrary, expression of the PPM2C mitochondrial gene was upregulated in our study. Protein phosphatase 1J (PPM1J_mouse, PP2C family) was found to be an *in vivo* substrate for calpain 3 [40].

In later stages of the disease, the muscle pathology is characterized mainly by the presence of lobulated fibers (LF), which are composed of misaligned myofibrils that form a lobular pattern, in addition to fiber size variation and interstitial fibrosis. Lobulated muscle fibers reflect an abnormal spatial distribution of the intermyofibrillar mitochondria network [51]. In C3KO mice, abnormal A-bands were seen, suggesting a role for calpain 3 in correct formation of sarcomeres or maintenance of sarcomere alignment [14].

mRNA expression profiles were specifically altered in LGMD2A muscles with lobulated fibers Keira et al [52]. Genes encoding for extracellular matrix (ECM)/membrane-related, cytoskeletal, or sarcomeric proteins were also upregulated in LF muscles.

According to these results, identification of these mitochondrial proteins suggests that CAPN3 may be involved in mitochondrial protein turnover.

### Common genes with altered expression in different muscular dystrophies

According to Table 2, LGMD2A can be characterized as an active fibrotic disease with suppressed muscle regeneration, since LGMD2A cases share upregulation of the extracellular matrix (ECM) components with congenital muscular dystrophy cases and share downregulation of the transcription factors with Duchenne muscular dystrophies.

In muscles from patients with Duchenne muscular dystrophy, upregulated genes were mostly those related to immune response, sarcomeric, ECM, and cell growth, whereas downregulated genes were associated to energy metabolism, transcription/translation, signaling, and proteasomes [32].

c-fos and c-jun proteins have been described as showing strong cytoplasmic expression related to the degeneration process occurring in Duchenne and Becker muscular dystrophies [53]. However our results contradict the previously published results and they showed a strong downregulation of c-fos and c-jun in our samples.

Recently Gan et al [54] reported that Dishevelled (Dvl) and c-Jun form a complex with β-catenin-T-cell factor 4 (TCF-4) on the promoter of Wnt target genes and regulate gene transcription. c-Jun mediates Dvl association with the functional TCF-β-catenin complex and functions as a key component of Wnt signaling *in vivo*. Since genes coding for proteins in this pathway are dysregulated in LGMD2A patients, it may be suggested that the downregulation of c-jun and other transcription factors observed in LGMD2A patients are regulated in an indirect way by calpain 3.

TFRC (transferrin receptor) and VLDLR (very low density lipoprotein receptor) are upregulated in LGMD2A patients as occurred in FSHD muscles [33]. Transferrin is a key myoblast trophic factor, initially promoting myoblast proliferation and subsequently supporting myogenic differentiation. However, TXNIP (thioredoxin-interacting protein) is downregulated in LGMD2A muscles and was also downregulated in FSHD samples [33]. Since TXNIP acts as an oxidative stress mediator, this finding is consistent with the enhanced vulnerability to oxidative stress seen in LGMD2A, as observed in FSHD myoblasts [33]. Many of the genes deregulated in facioscapulohumeral muscular dystrophy (FSHD) are involved in myogenesis, cell differentiation, and cell-cycle control.

According to the available information, it could be suggested that FSHD shared the greatest quantity of differentially expressed genes and the deregulation tendencies (up/downregulation) are the same and in a similar range of variation. However it would be difficult to establish any correlation given that in the FSHD, even in patients with the same deletion fragment, high variability of impairment and of muscle affectation grade is observed. Therefore, the data depend enormously on the place and on the moment that biopsy has been taken.

S100A6 (calcyclin) and S100A8 (calgranulin A), dysregulated in LGMD2A muscles, are involved in various intracellular and extracellular regulatory activities [55]. Upregulation of S100A6 expression was seen also in LGMD2B and as in other muscular dystrophies, the structural defect causes a general membrane instability that leads to an altered uptake of calcium ions into the muscle fibers [31]. Since calpain 3 interacts with dysferlin and AHNAK, a role of calpain 3 in membrane homeostasis has been suggested [8], [9]. The increased Ca2+ concentration probably influences expression of various signaling molecules whose transcription is sensitive to calcium concentration.

Dysferlin was not abnormally regulated in LGMD2A patients in the microarray study. The value obtained for the expression of the DYSF gene did not fulfil the established criteria to be considered as differently expressed (data not shown).

In this study mRNA levels are analysed, not protein quantities. Even if the protein is reduced in the Western Blot, this reduction may not be regulated at a transcriptional level, it may happen at a post-translation level. Since calpain 3 was shown to be in complex with dysferlin and it has been demonstrated that AHNAK, a novel component of the dysferlin protein complex, serves as a direct substrate of calpain 3 in cell culture, the lack of one of these proteins may justify the reduction of the other.

As described in previous works [31] while western blotting tests showed a reduction or the absence of dysferlin protein in most LGMD2B patients, the microarray data showed a reduction of dysferlin mRNA for some of the patients analysed. This could be due to the different types of mutations of the gene that affects the translation efficiency of the mRNA or the stability of the protein. Additionally, while protein analysis usually shows an absence of protein in the C57BL/10.SJL-*Dysf* mice, the microarray data did not reveal a reduction of dysferlin mRNA indicating that this primary genetic defect cannot be identified by expression profiling [37]. Moreover, it has been observed that neither calpain-3 nor caveolin was consistently reduced in dysferlinopathies.

The vast majority of upregulated genes in Fukuyama-type congenital muscular dystrophy (FCMD) and laminin-a2 deficient congenital muscular dystrophy (MDC1A) encode extracellular matrix components, presumably related to fibrotic change. However, mature muscle components were extremely downregulated in congenital muscular dystrophies [34].

Muscle regeneration is also a process that depends on the skeletal muscle basement membrane. Basement membrane is thought to not only maintain cell integrity but also to mediate signal transmission in cell differentiation, growth, attachment, survival, polarity, proliferation, and apoptosis [56], [57]. It is hypothesized that upregulation of ECM genes might arise from signal transduction defects due to basement membrane dysfunction. It is possible that muscle fibers keep high transcription levels of ECM to create basement membrane components [34].

Costameric proteins can interact with many components of both the sarcolemma and cytoskeleton. Different publications support a role for the costamere/Z-disk axis in mechanotransduction, the dynamic process through which mechanical stimuli are sensed by muscle cells and converted into biochemical responses [57].

Based on our results and since collagens, melusin and fibronectin, were deregulated, we may hypothesize that upregulation of ECM genes found in LGMD2A patients may result from signal transduction defects due to basement membrane dysfunction. Calpain 3 recognizes a wide range of substrates, including cytoskeletal proteins and myofibrillar proteins [40], [46]. These cytoskeletal proteins and matrix proteins contribute to cell shape, mechanical resistance, and morphological integrity of muscle cells, and are part of a complex network of filaments and tubules that transmit mechanical and chemical stimuli between cells. The cytoskeleton is not only involved in cell stability and integrity, but also plays a significant role in signal transmission from the cell membrane to the nucleus.

### LGMD2A and eosinophilic infiltrations

Presence of eosinophilic cells has recently been detected in muscle tissue from patients with mutations in the CAPN3 gene [16].

In our study, IL-32 was upregulated in LGMD2A patients with eosinophilic infiltrates (Table 3). Although IL-32 does not share sequence homology with known cytokine families, IL-32 induces various cytokines, human TNFα, and IL-8 in THP-1 monocytic cells. IL-32 activates typical cytokine signal pathways of nuclear factor-kappa B (NF-κB) and p38 mitogen-activated protein kinase [58]. The neutrophil-derived proteinase 3 (PR3) was identified as a putative IL-32 receptor [59], supporting the possibility that IL-32 upregulation in muscle may be chemoattractant for eosinophilic cells.

Eosinophilia has also been reported as a prominent feature of the necrotic phase in dystrophin-deficient mdx mice. This study suggested that eosinophilia was promoted by at least perforin-dependent cytotoxicity of effector T cells and T-cell production of interleukin-5 (IL-5) [60]. However these authors concluded that some eosinophilia of mdx muscle is independent from perforin-mediated processes and that it may be suggested that a similar mechanism of calpain 3 could act in this process.

Inflammatory features may be seen in some muscular dystrophies, such as facioscapulohumeral muscular dystrophy [17] and dysferlinopathies [19]. Moreover, a Becker muscular dystrophy presenting eosinophilic inflammatory myopathy was described by Weinstock et al [18].

Although the comparison between gene expression profiles between LGMD2A with and without eosinophilia would be interesting, it was not possible to perform. The methods used needed a higher quantity of samples to obtain significant results. Moreover, when a PCA plot was performed for LGMD2A patients only (including cases with and without eosinophilia), no different groups were created and this may be due to the low sample number too.

It seems that the comparison between asymptomatic patients with eosinophilia with control samples is more indicated to shed some light onto the initial mechanism that triggers the eosinophilic cell attraction to muscle. The comparison between asymptomatic patients and controls allows a clearer view due to a lower interfering expression variation.

In a first approach asymptomatic cases were considered as affected and were included into the patient group in the general analysis. These cases were included in the affected group due to their abnormal muscle biopsy pattern. Additionally reinforcing this decision, the PCA plots clustered together the LGMD2A case with or without eosinophilia.

Finally in an additional analysis, however, it was decided to consider them as a different group compared to the controls in order to obtain information about eosinofilic attraction in the early stage of the disease.

### Conclusions

In conclusion, upregulated genes were mostly those related to extracellular matrix, cell adhesion, sarcomeric proteins, and signal transduction. It is therefore suggested that different proteins located at or participating in the costameric region are involved in processes regulated by calpain 3 during skeletal muscle development. Upregulation of these proteins may indicate a compensatory attempt of the muscle, and since most of these genes are also upregulated in other dystrophic processes, upregulation might be relatively nonspecific.

It was also found that genes participating in the ubiquitin proteasome degradation pathway are deregulated in LGMD2A patients, which suggests that regulation of this pathway may be under the control of calpain 3 activity.

Finally, the upregulation of IL-32 and immunoglobulin genes may cause the eosinophil chemoattraction observed in the inflammatory findings in presymptomatic stages. This upregulation seems to disappear when the disease progresses. However, they might be quite specific markers for the disease.

Though samples taken from different muscles could add variability to the results of the expression array analysis, correlation of the results with the quantitative RT-PCR results gave strength to the findings. Gene expression profiling is presented as a useful approach to mine new data and hopefully open new perspectives for muscular disorders, shedding some light on identification of novel therapeutic targets for limb-girdle muscular dystrophies.

Looking ahead, each of these methods should be individually analyzed in the animal model and in cell models.

This analysis gives a total of 24 genes that may be considered as potential diagnostic or evolutionary biomarkers of the disease. However, this question will not be solved until the predictive value of these markers is proved in a series of patients with different evolutive status and secondly until the consistency of the results in different muscles and different laboratories is proved.
